# Radiological features of Adenomatoid odontogenic tumor: Report of a maxillary case and a mandibular one

**DOI:** 10.1002/ccr3.5301

**Published:** 2022-01-25

**Authors:** Imen Chaabani, Jed Bouguila, Rym Kammoun, Raja Chebbi, Badreddine Sriha, Habib Khochteli, Touhami Ben Alaya

**Affiliations:** ^1^ Department of Radiology University Dental Clinic Monastir Tunisia; ^2^ Maxillofacial and Aesthetic Surgery La Rabta Academic Hospital Tunis Tunisia; ^3^ Laboratory of Histology and Embryology Faculty of Dental Medicine University of Monastir Monastir Tunisia; ^4^ Department of Functional Exploration, Pain and Orofacial Dysfunction University Dental Clinic Monastir Tunisia; ^5^ Department of Anatomopathology University Hospital Farhat Hached Sousse Tunisia; ^6^ Department of Maxillofacial Surgery Faculty of Medicine of Sousse University Hospital Sahloul University of Sousse Sousse Tunisia

**Keywords:** Adenomatoid odontogenic tumor, dentigerous cyst, tomography, unerupted tooth, X‐Ray computed

## Abstract

We present two cases of AOT, the first case concerns a 23‐year‐old patient with an AOT located in the maxilla and the second case involves a 37‐year‐old patient presenting an AOT with mandibular localization.

## Graphical abstract

Adenomatoid odontogenic tumor is frequently misdiagnosed as a dentigerous cyst. However, it has a characteristic radiological appearance, consisting in a mixed image having a mostly liquid content and a peripheral calcification lining the wall. However, this aspect is often not detected on radiographs if the calcifications are too discreet.

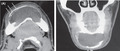

## 1 INTRODUCTION

Adenomatoid odontogenic tumor (AOT) is a rare benign tumor accounting for 3%of all odontogenic tumors. It was first described by Dreiblard in 1907 as a pseudo‐adenoameloblastoma.

At first, it was considered as a variant of ameloblastoma, from which it gained its previous name adenoameloblastoma. In 1948, Stafne considered it as a distinct pathologic entity.^1^

It was only in 1969 that Philipsen and Burn presented a review involving 76 cases, in which they showed that AOT is a well‐defined entity, distinct from solid or multi‐cystic ameloblastoma, and they therefore suggested the current term AOT.^1^, ^2^, ^3^

Its clinical symptomatology often includes a painless slow‐growing bone swelling.

The follicular variant associated with an impacted tooth is the most frequent type. It is therefore misdiagnosed as a dentigerous cyst, an ameloblastoma, or a kerato‐cystic odontogenic tumor.

The presence of intra‐lesional calcifications is considered as a characterizing distinctive element of AOT, allowing to have a differential diagnosis from other cystic bone lesions. Those calcified deposits are observed in approximately 78% of AOT.^4^

Thus, searching for these calcifications in imaging finds its importance to help guide diagnosis. The aim of this work was to describe the clinical, radiological, and anatomopathological characteristics of this rare tumor, to discuss its differential diagnosis and especially to highlight the benefit of radiological examinations in its diagnosis.

## 2 CASE REPORTS

### 2.1 Case 1

A 23‐year‐old patient presented with a painless nose and jaw swelling in the right side of the face that was progressing for 1 year. The patient's questionnaire revealed no previous extraction, traumatism, or infection. Extra‐oral examination revealed the presence of nose and jaw swelling in the right side of the face.

The integument had a normal aspect with no pain. Intra‐oral examination showed a vestibular swelling extending from the apical region of the right maxillary central incisor (11) to the right second premolar (15) with a normal mucosa.

On palpation, the swelling was depressible and painless. Dental examination revealed the absence of the right maxillary lateral incisor (12) on the arch as well as the persistence of the right temporary lateral incisor (52).

The maxillary right central incisor (11) was in vestibular position with a light mobility, and the remaining teeth were healthy. Diagnosis of a cystic or benign tumoral lesion having a cystic nature was made based on the absence of the right maxillary lateral incisor (12) on the arch and the presence of a painless and depressible slow‐growing swelling.

**FIGURE 1:**
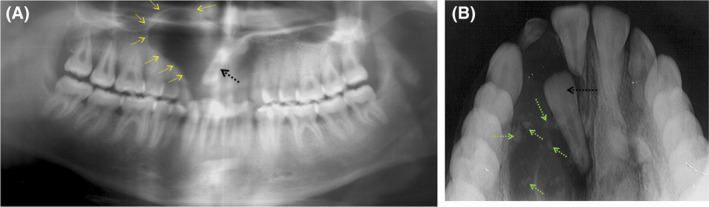
A, Panoramic radiography showing the presence of a cystic image associated with impacted 12. B, Maxillary occlusal bite showing the presence of a mixed image deforming the external cortex, the presence of impacted 12 and micro‐calcifications scattered within the image, with a root resorption of tooth 11

Panoramic radiograph showed a well‐defined radiolucent image with a peripheral condensation border having an oval shape, occupying the right median and paramedian region, extending upward to the apical region of tooth15 and fitting downward between the right maxillary central incisor (11) and the right maxillary canine (3) (Figure 1A).

This aspect reminds of an inverted peer shape. The right maxillary lateral incisor (12) was impacted, and it appeared to have a close rapport with this image. A light axis change of the right maxillary canine (13) resulting from the effect of the lesion extension was also noted (Figure 1A). Through the clinical and radiological data, various diagnoses were possible, notably a pericoronal cyst in relation to the impacted right maxillary lateral incisor (12) and a globulo‐maxillary cyst given the inverted peer shape and the insertion between the right maxillary lateral incisor (12) and the right maxillary canine (13) of the image.

Yet, diagnosis of cystic ameloblastoma remains possible. In order to analyze the lesion extension in the horizontal plane, an occlusal bite radiograph was performed. It showed posterior extension of the lesion, root resorption of the maxillary right central incisor (11), impaction of the right maxillary lateral incisor (12), and persistence of the right temporary lateral incisor (52).

A new essential element, consisting in the presence of micro‐calcifications within the radiolucent image, appeared (Figure 1B).

The diagnoses already suspected on the panoramic radiograph were therefore eliminated with the presence of this mixed aspect in the radiological image.

Thus, our diagnosis was oriented toward a benign tumoral lesion having a cystic nature, most likely presenting peripheral calcifications. These characteristics are the same as those described in calcifying odontogenic cysts, or AOT.

Faced with this uncertainty of diagnosis, computed tomography was performed. It revealed a well‐defined hypodense image with fluid nature, extending upward to the nasal fossa floor, downward to the apical region of the right first premolar (14) and the right second premolar (15), and fitting in the space between the right maxillary canine (13) and the right maxillary central incisor (11). Medially, the image extended up to the inter‐maxillary suture. Laterally, the external cortical bone was repressed and thinned without perforation.

**FIGURE 2:**
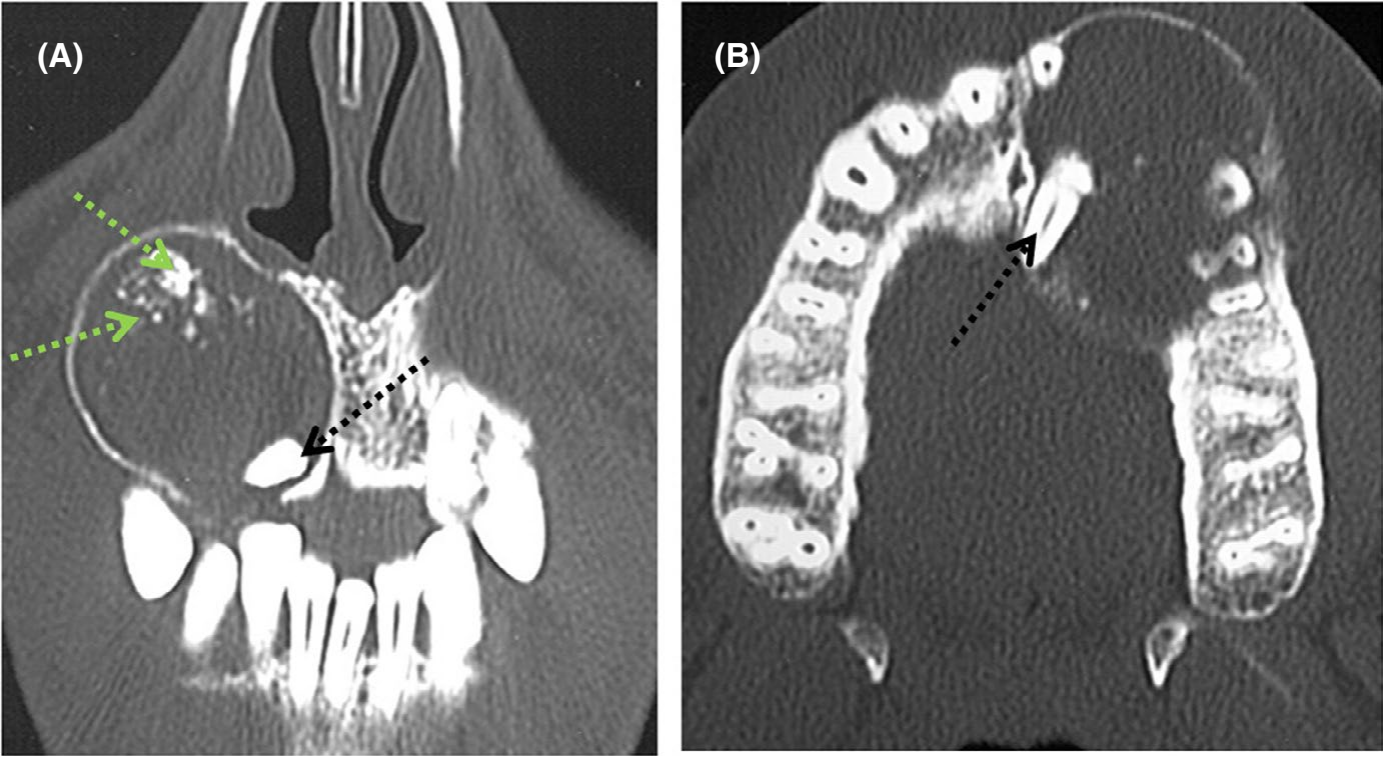
A, CT scan coronal slice in wider window revealing the presence of a mixed image pushing the external cortex, the bone palate, and the nasal fossae floor which are thinned, associated with the impacted 12 in full lesion. B, A CT scan axial slice showing a mixed image deforming the external and internal cortices, encompassing the apices of 11, 13, 14, and 15 with the presence of tooth 12 in full lesion

Within this image, impaction of the right maxillary lateral incisor (12) and the presence of peripheral intra‐lesional micro‐calcifications were confirmed (Figure 2). These findings pushed toward the two previously mentioned diagnoses, namely a calcifying odontogenic cyst or AOT.

This patient was operated under local anesthesia through a vestibular access. The lesion was totally enucleated with extraction of both the right temporary lateral incisor (52) and the impacted right maxillary lateral incisor (12).

Intra‐operatively, it was a well‐encapsulated lesion with fluid content. The clinical and radiological evolution after two weeks, one month, and one year were favorable without any sign of postoperative secondary infection or recurrence. Macroscopically, the anatomopathological examination showed that it was a cystic cavity comprising an impacted tooth. The cystic wall had variable thickness and presented budding areas. Histologically, the cavity contained eosinophilic material mixed with rare cellular debris. The budding areas of the wall were due to the presence of nodules formed of cylindric epithelial cells with regular nuclei arranged in rosette or in a pseudo‐glandular structure. In some locations, these cells formed anastomosing spans of isolated edematous connective tissue having a plexiform appearance.

Outside these cell areas, the wall was bordered by few rows of squamous cells resting on a strip of fibrous tissues having variable thickness. Finally, diagnosis of AOT was retained.

### 2.2 Case 2

A 37‐year‐old patient with no particular pathologic antecedents presented for mandibular swelling of the symphysis and the right parasymphysis regions that was evolving for several years.

The patient's questionnaire revealed no particularity. No previous extraction or infectious episodes were noted. On palpation, a painless vestibular filling of renitent consistency was noted. The lingual cortex showed no deformity. Dental examination revealed the absence of the right mandibular canine tooth (43) on the arch. The lip and chin sensation was preserved. The teeth in relation to the swelling had a negative vitality test without being mobile.

A pericoronal cyst in relation to the impacted right mandibular canine (43) was the suspected diagnosis given the absence of the tooth 43 on the arch without the notion of prior extraction.

**FIGURE 3:**
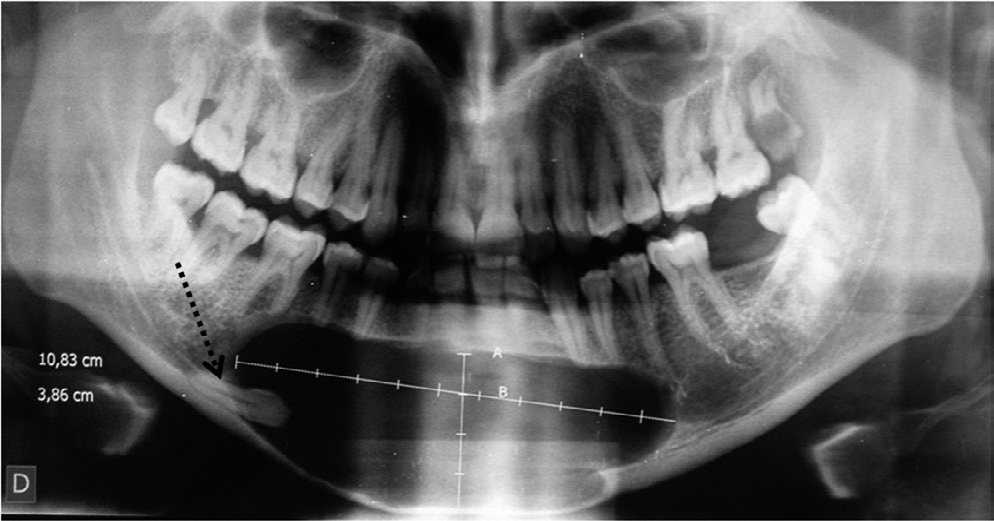
Panoramic radiography showing a cystic image occupying the entire symphyseal region and extending to the right and left parasymphyseal regions, associated with an impacted 43 in horizontal position close to the basilar border

Panoramic radiograph revealed a well‐defined homogenous radiolucent image with clear contours measuring approximately 11 cm × 4 cm of large diameter and extending from the mesial root of the right mandibular first molar to the root of the left mandibular second premolar (35). It had a close rapport with the apices of the overlying mandibular teeth. Downward, it extended toward the basilar border that was thinned (Figure 3).

Distally, the right mandibular canine (43) was impacted in horizontal position and repressed toward the basilar border. The border of the image at this level was in relation to the crown and to a part of this tooth root.

**FIGURE 4:**
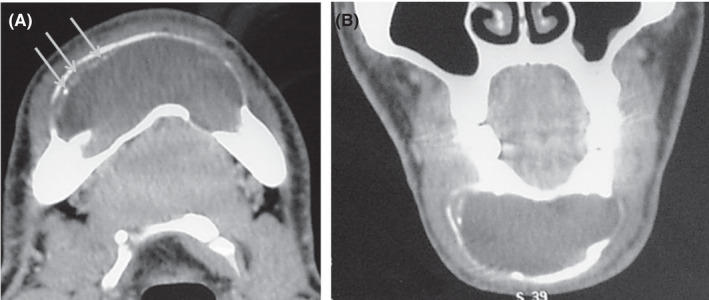
A, CT scan axial slice in narrow window showing the presence of a mixed image deforming the external and internal cortices which are thinned, with the presence of fine intra‐lesional calcifications lining the wall. B, A CT scan coronal slice showing the presence of fine intra‐lesional calcifications

At this stage, diagnosis of a pericoronal cyst in relation to the impacted right mandibular canine (43) was made. In order to establish a more accurate assessment of extension, computed tomography scan (CT) was performed. It confirmed the presence of hypodense image of fluid density repressing and essentially thinning the vestibular cortex. In certain sites, the lingual cortex was slightly thinned. The crown of the impacted 43 was encompassed by this image. A new element of semiology, consisting in the presence of fine calcifications arranged in periphery at the level of the vestibular wall, appeared on CT scan examination (Figure 4).

This radiographic aspect eliminated the previously made diagnosis of a pericoronal cyst. The most probable diagnosis was calcifying odontogenic cyst and AOT.

Preoperative endodontic treatment on the necrotic teeth was performed. The treatment was conducted under general anesthesia, and it involved the lesion excision through a vestibular access and extraction of the impacted right mandibular canine (43).

**FIGURE 5:**
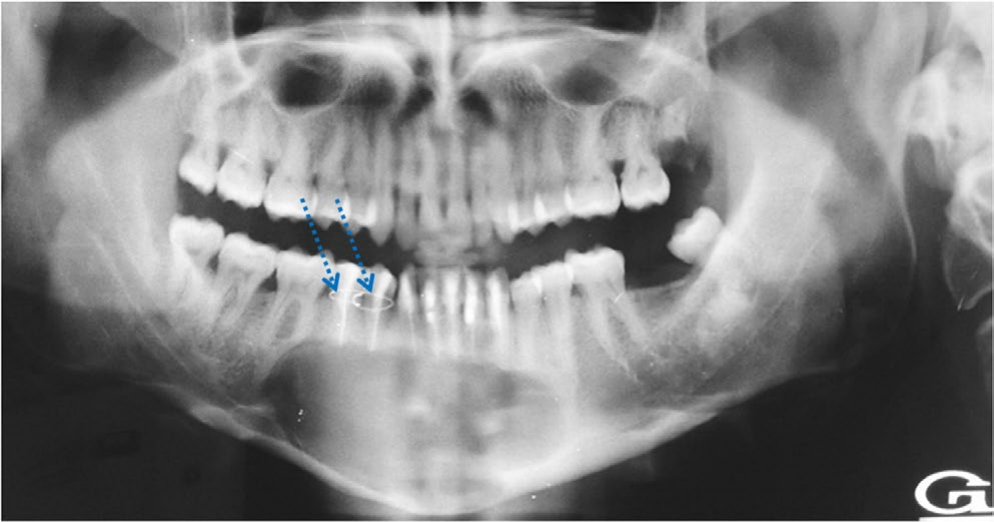
Postoperative panoramic image showing the excision site, the canal obturations of teeth starting from tooth 45 to 33, and the metal wire suture of 45 to 44

Preoperatively, the right mandibular second premolar (45) was mobile. It was therefore attached to the right mandibular first premolar (44) using a steel wire suture. Postoperative follow‐ups were simple (Figure 5).

The panoramic radiograph performed two months postoperatively showed that the excision site presented a beginning of reossification. In the last follow‐up, six months after the intervention, no signs of superinfection or recurrence were observed.

Macroscopically, the anatomopathological study showed an elastic grayish cystic wall having a smooth outer surface, comprising grayish vegetations and measuring 0.2 to 1 cm of large axis. The internal surface was covered with a hemorrhagic coating.

**FIGURE 6:**
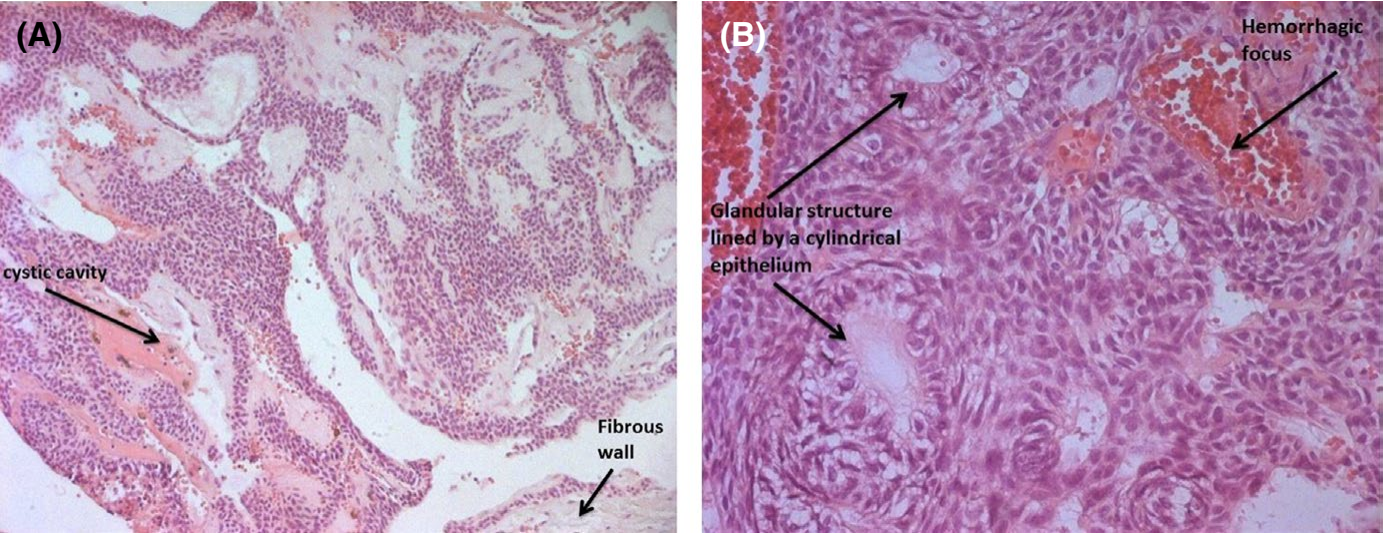
Histological aspect of AOT. A, Hematoxylin‐eosin × 100. B, Hematoxylin‐eosin × 200

Histologically, the cavity presented a fibrous wall dissociated by hemorrhagic suffusions and bordered by focally ulcerated epithelium. At the level of vegetations, the epithelium showed proliferation of oval cells with clear cytoplasm and regular nucleus, associated, in certain sites, with cylindric cells having eosinophilic cytoplasm organized in excavated massive of cavities and enclosing focally calcified eosinophilic material (Figure 6).

Hence, diagnosis of an AOT was made.

## 3 DISCUSSION

In the latest classification of the World Health Organization (2017), AOT is considered as a tumor integrated in the group of epithelial odontogenic tumors.^5^ It accounts for only 1% of maxillary cysts and tumors and 3% of odontogenic tumors. It occupies the fifth position after odontoma, cementoblastoma, myxoma, and ameloblastoma. It is more frequent in females than in males with a sex ratio of 2:1, and it is mainly observed during the second decade of life.

The lesions are more prevalent in the maxilla than in the mandible, and it is more common in the anterior region than in the posterior region.^6^, ^7^, ^8^

In our two reported cases, the localization of AOT perfectly corresponds to the data available in the literature both for maxillary and mandibular localization. AOT usually measures 1.5 to 3 cm of large axis, but it can sometimes reach a more important volume that can be responsible for dental displacements as well as functional and/ or esthetic disorders.

In the second reported case, the tumor reached a very important volume, measuring approximately 11 cm × 4 cm of large axis, resulting in necrosis of the involved teeth and a troublesome jaw swelling that was the major motive for the patient to seek treatment.

In fact, the size of the lesion is correlated with increasing aging and it is significantly associated with features, such as increased root resorption, ill‐defined borders, expansion, cortex perforation, and lesions crossing the midline. These changes may reflect a longer duration of the lesion due to late diagnosis.^9^

Three clinic‐topographic variants of AOT are identified: Follicular, extra‐follicular, and peripheral.

The follicular variant is the most frequent. It is an intra‐osseous lesion associated with an impacted tooth. Our two reported cases correspond to this type (an impacted right maxillary lateral incisor (12) in the first case and an impacted right mandibular canine (43) in the second one).

A large study including 105 cases of AOT demonstrated that in 51 cases (48.5%), tumors were located in the anterior maxilla, representing the most common anatomic site, followed by 31 cases (29.5%) in the anterior mandible, 16 cases (15.2%) in the posterior mandible and 7 cases (6.6%) in the posterior maxilla.^10^

The extra‐follicular or extra‐coronal variant is intra‐osseous having no relation to an impacted tooth. Philipsen suggested four sub‐types for this variant, classified from E1 to E4.

E1: tumor without any relation to the tooth.

E2: tumor between two teeth roots.

E3: tumor encompassing the tooth root apex.

E4: tumor encompassing an intermediate part of the tooth root.

The peripheral type which is extra‐osseous is localized at the gingival tissue. Clinically, it usually appears as a labial gingival mass overhanging the crown (93% of peripheral lesions).^11^, ^12^

This extra‐osseous variant is exclusively restricted to the anterior maxillary labial gingiva, with only 0.3% of cases being reported in the mandible.^5^ Moreover, the most common tooth involved in follicular AOT is the permanent canine, with maxillary canine accounting for 40% of cases and unerupted third molars representing 2.8% of cases. However, in the extra‐follicular variant, the permanent canine accounts for 89% of cases.^7^ Permanent incisors, premolars, molars, and deciduous teeth are rarely involved.^13^

The study conducted by T Becker et al. showed that the extra‐osseous peripheral variant is the least frequent (5% of the studied cases). The intra‐osseous variant of the follicular type is more frequent on the maxilla; however, the intra‐osseous variant of the extra‐follicular type is more frequent on the mandible.^9^

The pathogenesis of AOT is still poorly known and uncertain, having various origins as mentioned in the literature. Yet, the resemblance between the cylindric cells and the ameloblasts, and the frequent association with impacted teeth suggest that the tumor could originate from the dental epithelium.^14^, ^15^

This could explain the possible radio‐clinical confusion between AOT and follicular cysts.

Adenomatoid odontogenic tumor is often discovered fortuitously, either during a routine dental examination or during a radiographic examination carried out for a check‐up (orthodontic treatment, delayed tooth eruption, etc.). Sometimes, it is the development of a slow‐progressing, often isolated and painless bone swelling that alerts the patient and incites him to seek treatment. This swelling can sometimes be accompanied by dental signs (displacement, mobility of the neighboring teeth, and delayed eruption).^7^

Therefore, the main clinical findings are asymptomatic lesions causing facial asymmetry as a result of cortical bone expansion and diffuse swelling in the buccal and palatal/lingual vestibules.^10^

When the lesion is intra‐osseous, it causes thinning, and even a minimal rupture of the cortex which can clinically manifest through a palpable and depressible swelling that could result in facial asymmetry.

In this form, it is difficult to clinically differentiate between AOT and other intra‐osseous benign lesions, such as dentigerous cysts, calcifying odontogenic cysts, and ameloblastomas.

In most cases, the content of the lesion is liquid. However, for extensive AOT, a solid consistency may be present in the walls.^16^

The distinction between AOT in its follicular form and dentigerous cysts is essentially based on the rapport of the image to the crown or the root of the tooth involved. AOT is rather suspected in case of an image encompassing the tooth crown and root. However, in case of dentigerous cysts, the image would rather involve only the crown.^2^, ^6^

Radiologically, intra‐osseous AOT presents a unilocular osteolytic image with well‐defined contours containing, in certain locations, discrete calcified foci. In the most advanced cases, more or less marked radio‐opaque foci can be found, constituting a useful diagnostic tool.

The extra‐osseous, peripheral, or gingival types of AOT are rarely detected radiographically; however, slight erosion of the underlying alveolar bone cortex may be present.^17^

When calcifications appear in imaging, diagnosis of a dentigerous cyst is ruled out. Our diagnosis was therefore oriented toward AOT, calcifying odontogenic cysts, or fibro‐cemento‐osseous tumors.

An essential clinical sign, namely cortical depressibility to palpation associated with elements of radiographic semiology, like fluid density at the CT scan examination and the peripheral location of the calcifications are in favor of diagnosing a cystic tumoral lesion, such as AOT or calcifying odontogenic cysts. In this case, calcifying epithelial odontogenic tumors and fibro‐osseous tumors are eliminated.^18^

These radiographic characteristics are the same as those observed in our two cases where intra‐lesional and peripheral fine calcifications lined the wall with fluid density at the scanner.

The distinction between calcifying odontogenic cysts and AOT can only be performed anatomopathologically. The anterior maxillary localization is in favor of AOT.^19^

For AOT, the calcifications found at imaging are described as spotted areas or snowflakes, calcified areas having unequal size, calcified speculated areas, fine and scattered radio‐opacities, irregular radio‐opacities, amorphous radio‐opacities, and fine and discrete radio‐opacities.

It is important to note that panoramic imaging is often insufficient to reveal the presence of these calcifications. However, intra‐oral radiographic techniques are more efficient thanks to their visualization.^4^, ^9^ Indeed, radiographic imaging does not show the presence of calcifications within the image which appears completely radiolucent, thus evoking the diagnosis of a cystic lesion.

Maxillary occlusal radiograph clearly showed the presence of fine radio‐opaque calcifications, which directed our diagnosis toward a tumoral lesion.

In fact, the perception of radio‐opacities in radiographs may depend on the amounts of calcifications and the radiographic techniques. Minimal calcifications (particularly a thin radio‐opaque line) might not be detectable on conventional images due to superimposition of the structures.^19^

Thanks to its high resolution and good spatial definition, sectional imaging (multi‐bar scanner or Cone Beam Computed Tomography) allows a better approach to the lesion density. The presence of intra‐lesional calcifications and their peripheral disposition on the image are well‐detected on the different slices. Sectional imaging is more efficient in the detection of these calcifications and in the analysis of their central or peripheral locations. The presence of these calcifications on the different slices eliminates the diagnosis of a cystic lesion, previously made based on standard radiographs.

Analysis of the lesional content allows a better diagnostic orientation, essentially through the possibility of density measurement provided by the scanner.^20^

With the technological evolution, cone beam computed tomography (CBCT) is currently used in all the fields of dental medicine and maxillofacial surgery. The main advantage of CBCT radiography is the multi‐planar cross‐sectional images in various orientations and the three‐dimensional reconstructions based on a single scan of the fields of view, varying from a single tooth to the whole maxilla‐facial area. CBCT imaging is superior to panoramic radiography with regard to elimination of superimposition and the excellent contrast resolution for mineralized tissue, such as teeth, bones, and calcified spots. Therefore, CBCT is advantageous in terms of demonstrating the detailed internal structures of lesions, particularly when the calcifications are minimal or the superimposition is serious in the maxillary region. Moreover, CBCT provides a better display of the extent and the complex spatial relationship of the lesions with the surrounding structures.^21^, ^22^

The diagnosis suspected based on clinical and radiographic data can only be confirmed by the anatomopathological study of the operative pieces.

Macroscopically, it is a cavity of 1 to 5 cm in diameter, having a wall of variable thickness and presenting some small endocavity over elevations. Histologically, the epithelial cells are arranged in spans or compact lobules within a small connective stroma. In certain sites, they can adopt an ameloblastic cylindric aspect with the presence of pseudo‐glandular cavities devoid of mucous secretions (PAS and negative blue Alcian).

Within the epithelial masses, small masses of eosinophilic hyaline material capable of being calcified are observed.

The histo‐enzymologic activities of these cells are particular. In fact, these cells have an intense ATPase and alkaline phosphatase activity on the cytoplasmic membrane.

In electronic microscopy, the rare cases studied so far have revealed two cell types: Some are polygonal or cylindric, rich in tonofilaments resembling preameloblasts, and others are smaller stellate cells recalling the structure of stratum intermediun and stellate reticulum of the dental bud.

Concerning the exact nature of hyaline deposits, it is controversial. Some authors assimilate it to an amyloid‐like substance close to the enamel matrix because it contains a rich network of microfibrils. However, for others, it is a dysplasic dentine comprising calcifications corresponding to apatite crystals.^8^, ^14^

On the therapeutic level, the treatment of choice consists in complete enucleation. In case of an impacted tooth in a favorable position associated with AOT, it can be preserved and placed on the arch using orthodontic means.

Evolution is constantly favorable. Recurrences are exceptional, and no malignant transformation is reported.^6^

## 4 CONCLUSION

The two cases are presented in great radiological details in an effort to share radiographic features that can assist pathologists and clinicians who, at some point, need to diagnose and treat patients with AOT.

In fact, AOT is a benign odontogenic tumor of the maxillae. The clinical aspect depends on the intra‐osseous or extra‐osseous form of the tumor. Histopathology can confirm diagnosis.

However, a characteristic radiological aspect, consisting in the presence of a mixed image with calcifications arranged in the periphery, may be in favor of this diagnosis.

## CONFLICT OF INTEREST

None.

## AUTHOR CONTRIBUTION

I. C, R.C, and T. A: contributed to the diagnosis of the cases, prepared, and drafted the manuscript; B.S and R. K: contributed to the anatomopathological diagnosis of the cases. J.B and H.K: contributed to the oral and maxilla‐facial surgery of the cases.

## ETHICAL APPROVAL

This article does not contain any studies with human or animal subjects performed by any of the authors.

## CONSENT

A written informed consent was obtained in accordance with the journal's patient consent policy.

## Data Availability

Data available on request from the authors.

## References

[1] Stafne EC . Epithelial tumors associated with developmental cysts of the maxilla; a report of three cases. Oral Surg Oral Med Oral Pathol. 1948;1:887‐894. 18888040 10.1016/0030-4220(48)90114-5

[2] Kallel R , Ayadi L , Gouiaa N , et al. Tumeur odontogène adénomatoïde extrafolliculaire: à propos de deux cas. Rev Med Brux. 2009;30(5):511‐514. 19998797

[3] Philipsen H , Reichart PA . Adenomatoid odontogenic tumour: facts and figures. Oral Oncol. 1999;35(2):125‐131. 10435145 10.1016/s1368-8375(98)00111-0

[4] Handschel JG , Depprich RA , Zimmermann AC , Braunstein S , Kübler NR . Adenomatoid odontogenic tumor of the mandible: review of the literature and report of a rare case. Head and Face Med. 2005;1(3):1‐5. 16270916 10.1186/1746-160X-1-3 PMC1266042

[5] Wright JM , Vered M . Update from the 4th edition of the World Health Organization Classification of head and neck tumours: odontogenic and maxillofacial bone tumors. Head and Neck Pathol. 2017;11(1):68‐77. 28247226 10.1007/s12105-017-0794-1 PMC5340735

[6] Chrcanovic BR , Gomez RS . Adenomatoid odontogenic tumor: an updated analysis of the cases reported in the literature. J Oral Pathol Med. 2019;48(1):10‐16. 30256456 10.1111/jop.12783

[7] Genno NK , Aoun N , El Toum S . Adenomatoid odontogenic tumor associated with an impacted maxillary lateral incisor: a case report with five‐year follow‐up. Case Rep Dent. 2017;2017:1709492. 29214083 10.1155/2017/1709492 PMC5682065

[8] Siriwardena B , Tennakoon T , Tilakaratne WM . Relative frequency of odontogenic tumors in Sri Lanka: analysis of 1677 cases. Pathol Res Pract. 2012;208(4):225‐230. 22439972 10.1016/j.prp.2012.02.008

[9] Becker T , Buchner A , Kaffe I . Critical evaluation of the radiological and clinical features of adenomatoid odontogenic tumour. Dentomaxillofac Radiol. 2012;41(7):533‐540. 22752319 10.1259/dmfr/19253953 PMC3608372

[10] Roza ALOC , Carlos R , van Heerden WFP , et al. An international collaborative study of 105 new cases of adenomatoid odontogenic tumors. Oral Surg Oral Med Oral Pathol Oral Radiol. 2021;132(3):327‐338. 32680811 10.1016/j.oooo.2020.06.001

[11] Farah‐Klibi F , Ferchichi L , BeyâaRassou H , et al. Adenomatoid odontogenic tumor: two cases. Rev Stomatol Chir Maxillo Fac. 2007;108(1):61‐64. 10.1016/j.stomax.2005.12.002 17275864

[12] Ide F , Mishima K , Kikuchi K , et al. Development and growth of adenomatoid odontogenic tumor related to formation and eruption of teeth. Head and Neck Pathol. 2011;5(2):123‐132. 21380723 10.1007/s12105-011-0253-3 PMC3098332

[13] Saini N , Kadian B , Rajain T , Narang S , Namdev R . Extra follicular adenomatoid odontogenic tumor in the maxillary incisor region disguised as gingival swelling. Contemp Clin Dent. 2020;11(2):184‐189. 33110335 10.4103/ccd.ccd_344_20 PMC7583531

[14] de Matos FR , Nonaka CF , Pinto LP , de Souza LB , de Almeida Freitas R . Adenomatoid odontogenic tumor: retrospective study of 15 cases with emphasis on histopathologic features. Head and Neck Pathol. 2012;6(4):430‐437. 22869356 10.1007/s12105-012-0388-x PMC3500904

[15] Thakur A , Tupkari JV , Joy T , Hanchate AV . Adenomatoid odontogenic tumor: what is the true nature? Med Hypotheses. 2016;97:90‐93. 27876138 10.1016/j.mehy.2016.10.024

[16] Patel HB , Movaniya PN , Desai NN , Makwana TR , Makwana KG , Mehta PD . Adenomatoid odontogenic tumor associated with impacted mandibular canine ‐ a case report. Annals Maxillofac Surg. 2020;10(2):484‐487. 10.4103/ams.ams_77_20 PMC7943982 33708601

[17] Garg D , Palaskar S , Shetty VP , Bhushan A . Adenomatoid odontogenic tumor ‐ hamartoma or true neoplasm: a case report. J Oral Sci. 2009;51(1):155‐159. 19325215 10.2334/josnusd.51.155

[18] Fregnani ER , Pires FR , Quezada RD , ShihIeM VPA , de Almeida OP . Calcifying odontogenic cyst: clinicopathological features and immunohistochemical profile of 10 cases. J Oral Pathol Med. 2003;32(3):163‐170. 12581386 10.1034/j.1600-0714.2003.00070.x

[19] Chindasombatjaroen J , Poomsawat S , Kakimoto N , Shimamoto H . Calcifying cystic odontogenic tumor and adenomatoid odontogenic tumor: radiographic evaluation. Oral Surg Oral Med Oral Pathol Oral Radiol. 2012;114(6):796‐803. 23159119 10.1016/j.oooo.2012.08.452

[20] Uehara K , Hisatomi M , Munhoz L . Assessment of Hounsfield unit in the differential diagnosis of odontogenic cysts. Dentomaxillofac Radiol. 2020;49:20200188. 10.1259/dmfr.20200188 PMC7860949 32783633

[21] Sadasivan A , Ramesh R , Kurien NM . Peripheral adenomatoid odontogenic tumor —A rare cause of gingival enlargement: a case report with CBCT findings. Clin Cosmet Investig Dent. 2020;21(12):297‐304. 10.2147/CCIDE.S261308 PMC7398881 32801923

[22] Jiang M , You M , Wang H , Xu L . Characteristic features of the adenomatoid odontogenic tumour on cone beam CT. Dentomaxillofac Radiol. 2014;43(6):20140016. 24940808 10.1259/dmfr.20140016 PMC4141673

